# A Challenge for a Unique Dengue Vector Control Programme: Assessment of the Spatial Variation of Insecticide Resistance Status amongst *Aedes aegypti* and *Aedes albopictus* Populations in Gampaha District, Sri Lanka

**DOI:** 10.1155/2021/6619175

**Published:** 2021-04-02

**Authors:** Rasika Dalpadado, Nayana Gunathilaka, Deepika Amarasinghe, Lahiru Udayanaga

**Affiliations:** ^1^Regional Director of Health Services Office, Gampaha District, Gampaha, Sri Lanka; ^2^Department of Parasitology, Faculty of Medicine, University of Kelaniya, Ragama, Sri Lanka; ^3^Department of Zoology and Environmental Management, Faculty of Science, University of Kelaniya, Dalugama, Sri Lanka; ^4^Department of Biosystems Engineering, Faculty of Agriculture and Plantation Management, Wayamba University of Sri Lanka, Makadura, Sri Lanka

## Abstract

**Background:**

To date, dengue is considered an important public health problem in Sri Lanka. Irrational use of insecticides without evidence-based applications has primed the development of resistance in mosquito vectors.

**Method:**

The present study investigated the resistance status of *Aedes aegypti* and *Aedes albopictus* to commonly used insecticides in three selected Medical Officer of Health (MOH) areas (i.e., Attanagalla, Dompe, and Negombo) in Gampaha District, Western Province of Sri Lanka. Entomological surveys were performed using ovitraps and larval collections. Larval bioassays were carried out to determine the LC_50_, LC_90_, and LC_95_ and susceptibility status for organophosphate temephos, whereas adult bioassays were performed to test the 0.03% deltamethrin and 0.8% malathion susceptibility.

**Results:**

The study revealed that the temephos concentrations required to control *Ae. aegypti* (13.7-17.7 times) and *Ae. albopictus* (4.6-7.6 times) are higher than the diagnostic concentration (0.012 mg/L) proposed by the World Health Organization. The highest resistance levels were observed for both *Ae. aegypti* (14 ± 1.87) and *Ae. albopictu*s (36 ± 1.87) collected from the Negombo MOH area. Therefore, the WHO recommended diagnostic concentration is no longer effective in controlling *Ae. aegypti* and *Ae. albopictus* larvae in these areas. Both the dengue vectors have evolved a high level of insecticide resistance to malathion and deltamethrin in the Gampaha District except *Ae. albopictus* mosquitoes in rural areas. Further, vectors in rural areas are indicated susceptible (>98%) to pyrethroids and emergence of resistance (<97%) for organophosphate insecticides.

**Conclusion:**

The results of this study warrant the vector management authorities on the proper application of insecticides and rational use in vector control. The susceptibility status of vector mosquitoes should be continuously monitored especially in dengue-endemic areas parallel to the routine surveillance programme. Further molecular studies are strongly recommended to determine the Knockdown Resistance (kdr) mutations among *Aedes* populations.

## 1. Background

Dengue is the most rapidly spreading mosquito-borne viral disease in the world, affecting more than 2.5 million people in urban and periurban areas in over 100 countries, especially in South and Southeast Asia [1]. *Aedes* (*Stegomyia*) *aegypti* and *Aedes* (*Stegomyia*) *albopictus* are the two most important vectors of dengue in the world [2]. In the South-East Asian region, *Ae. aegypti* is considered the principal epidemic vector of the dengue virus, while *Ae. albopictus* is an endemic vector, also contributing to the viral transmission [3].

In Sri Lanka, dengue was serologically diagnosed for the first time in 1960 and the first time as an allochthonous case in 1962 [4, 5]. Since early 2000, progressively larger epidemics of dengue with more cases of Dengue Hemorrhagic Fever (DHF) occurred at regular intervals. A major upward shift to a high incidence of dengue has been reported since 2009. For the last 5 years, approximately 47-55% of dengue cases have been reported in the Western Province of Sri Lanka and the second-highest number of dengue cases was reported from the Gampaha District [6] which is considered to be the most populated residential district in Sri Lanka.

Effective vaccines and therapies for most human diseases caused by arboviruses including dengue disease are still under a developmental stage [7]. Therefore, suppression of vector population and limiting the vector-human contact is the most reasonable way to control vector-borne diseases like dengue, because complete eradication of suitable vectors and elimination of pathogen or parasite is rather unrealistic through control approaches [8, 9].

Currently, vector control approaches are mainly based on source reduction, application of insecticides, public health education, and legislations [10–12]. The use of insecticides is considered the most efficient application in vector control programmes. Insecticide applications can thereby vary from aerosol-space spraying, coils, lotions, clothes, or curtains embedded with certain active insecticide compounds and mass fogging to the usage of larvicides in breeding waters [9]. Thermal fogs have been widely used for the control of *Ae. aegypti* and *Ae. albopictus* in Sri Lanka for several years especially during dengue outbreak situations [10].

The most commonly used insecticides in dengue control in Sri Lanka include technical malathion (organophosphate), Pesguard FG 161® (pyrethroids), temephos (organophosphates), and *Bacillus thuringiensis israelensis* (Bti-bio-pesticide). The technical malathion 95% ULV and Pesguard FG 161 are used as adulticide and temephos 1% SG, temephos 50 EC, and Bti use as larvicides in vector control [13]. Consequences of national policy interruption by the usage of massive insecticide-based controls with the same active compounds may cause developing resistance in vectors against the insecticides. Therefore, the use of insecticides to control mosquitoes should always be in line with insecticide resistance monitoring and management, which is a neglected component in vector control efforts.

Detailed evidence of insecticide resistance status of dengue vectors in Sri Lanka has been limitedly documented, and the effectiveness of some insecticides used for vector control is unknown. Investigations on the resistance status to the insecticides at the regional level are essential for effective chemical-based vector control interventions. The only documented study in the Gampaha District, Sri Lanka, has been conducted at a single location to represent the whole district. It has indicated that both *Ae. aegypti* and *Ae. albopictus* have evolved resistance to currently used insecticides in Sri Lanka [10, 14]. However, such generalized data as evidence for vector control may not apply to the whole district. During the last five years, approximately 400 kg of technical malathion and 400 L of pyrethroid insecticides have been used each year for thermal fogging activities in the Negombo MOH area while usage of both organophosphates and pyrethroids was below 50 kg in rural MOH area like Dompe [15]. Therefore, the present study was conducted to provide the first descriptive figures to the insecticide resistance status of dengue vectors representing the urban, suburban, and rural populations in the district of Gampaha, Sri Lanka. This would facilitate in decision-making, implementing effective, economical, and sustainable dengue vector control measures in the district with the rational use of insecticides.

## 2. Methods

### 2.1. Study Area

**Figure 1:**
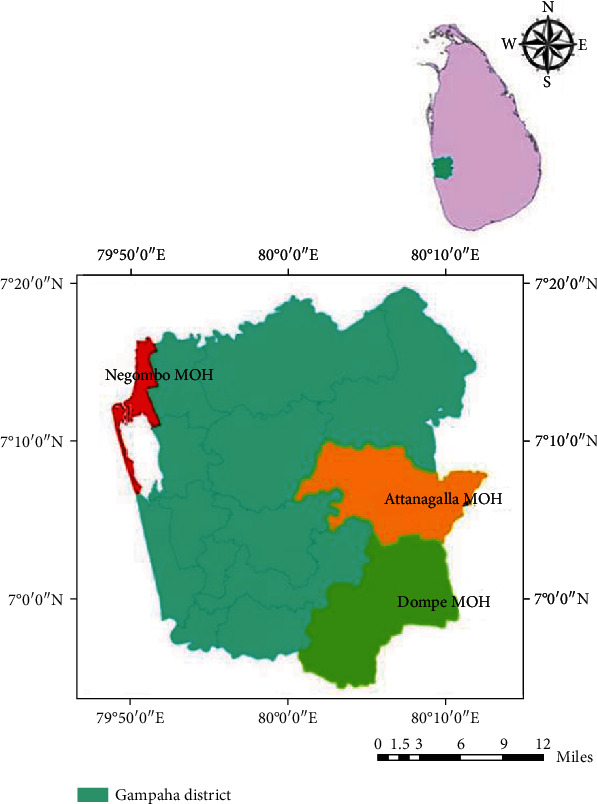
Location of the study areas in the Gampaha District, Sri Lanka.

The study was conducted in Gampaha District, in the Western Province of Sri Lanka, which records the second-highest number of dengue cases over the last two decades. It is being considered the highest residential population in Sri Lanka. The district extends over 1,387 km^2^ and has a population density of approximately 1,800. Three Medical Officer of Health (MOH) areas, namely, Attanagalla (7°05′60.00^″^N: 80°06′60.00^″^E), Dompe (6°56′25.42^″^N: 80°4′37.91^″^E), and Negombo (7°12′60.00^″^N: 79°49′59.99^″^E), were selected for the present study representing rural, suburban, and urban settings, respectively (Figure 1).

In the selection of study areas, geographic areas that are located inside towns and cities were described as urban whereas rural describe geographic areas that are located outside towns and cities, usually less developed with significant land cover under agriculture and/or natural vegetation. Areas with mixed characteristics were considered suburban [16].

### 2.2. Collection of Mosquitoes

*Aedes* eggs were collected from December 2016 to July 2019 at two-month intervals from all selected study sites using ovitraps. Ovitraps were prepared using black plastic cups of 250 mL capacity with filter papers as oviposition substrates. A total of 100 ovitraps was placed outdoor and indoor in randomly selected 50 houses in each locality. The positive ovitraps were collected after 5-7 days, and eggs were reared in the laboratory.

In each selected study area, larval and pupal collections were conducted every month by random sampling. A sentinel location was identified by selecting a house randomly each month. A minimum of 100 houses was surveyed within a radius of 200-300 m at the sentinel site selected. Larvae and pupae (F0 generation) were collected from permanent/temporary domestic and peridomestic breeding locations encountered in each selected area, separately. The live specimens were transferred into larval rearing vials and transported safely to the insectary at the Department of Parasitology, Faculty of Medicine, University of Kelaniya, Ragama, Sri Lanka.

### 2.3. Establishment of Adult Mosquito Colony

The immature stages collected from three study areas were reared at separate rearing cages under confined laboratory conditions (27 ± 2°C; 75 ± 5% relative humidity (RH); 12 : 12 [L : D] h photoperiod) with the recognition of the site collected. The larvae were fed with a specified larval diet formula described before [17, 18]. Emerged mosquitoes were identified to the species level and reared separately at different cages according to the place of origin (urban, semiurban, or rural). The adult female mosquitoes were housed at mosquito rearing cages (24 × 24 × 24 cm^3^) with mesh screening on top, provided with a 10% sugar solution and water *ad libitum* twice a day (morning and evening).

### 2.4. Larval Susceptibility Test

Larval bioassays were conducted using WHO standard susceptibility test kits provided by the National Dengue Control Unit following the WHO protocol [12] to determine the efficacy of temephos (organophosphate). Concentrations of test solutions (0.00625 mg/L, 0.0125 mg/L, 0.025 mg/L, 0.0375 mg/L, and 0.05 mg/L) were prepared using 3.125 mg/L of temephos stock solution.

A batch of 20 field-collected larvae (III and IV instar stages) was introduced to each freshly prepared test solution series separately. The larval mortality was recorded after 24 hours of insecticide exposure. Larval bioassays for each concentration were repeated five times with control trials using the mosquitoes collected from each MOH separately to determine lethal concentrations (LC) which result in 50%, 90%, and 99% mortality (LC_50_, LC_90_, and LC_99_). Water temperature was maintained (25°C ± 2°C) throughout the investigation. Moribund larvae were also added to dead larvae for calculating percentage mortality, and pupated larvae were discarded during the test. The tests were conducted separately for *Ae. aegypti* and *Ae. albopictus* collected from each selected study area.

### 2.5. Adult Susceptibility Assay

Three- to five-day-old female adult mosquitoes of *Ae. aegypti* and *Ae. albopictus* were selected for the adult bioassays according to the site of collection separately. The susceptibility was performed following standard guidelines of the WHO with tarsal contact exposure to the impregnated papers with insecticides in the standard kit [12]. Susceptibility level to 0.03% deltamethrin and 0.8% malathion was evaluated. A batch of 20 mosquitoes was exposed to the insecticide-treated paper line around the bioassay chamber, and the number of knock-down mosquitoes was recorded after the one-hour exposure period.

The surviving mosquitoes were transferred to the holding tubes and fed with 5% sucrose solution. Mortality was enumerated after 24 hours of exposure, and the mortality rate was calculated. The experiment was repeated five times for each chemical and mosquito species collected from different locations with a control group on each occasion. If mosquito mortality in the control was exceeded 10%, the corrected mortality was calculated using Abbott's formula [19]. The tests were rejected if the corrected mortality in the control exceeded 10%.

### 2.6. Data Analysis and Interpretation

The mortality levels were defined as susceptible (>97% mortality), the emergence of possible resistance (90-97% mortality), and resistant (<90% mortality) according to the susceptibility criteria defined by the WHO guidelines for Monitoring and managing insecticide resistance in *Aedes* mosquito populations [12]. When calculating larval mortality, test results were discarded if more than 10% of larvae pupated, while the control mortality was maintained between 5% and 20%. In all the cases, corrected percentage mortality was calculated using Abbott's formula [19]. Similarly, during adult bioassays when adult mosquito mortality in the control tubes exceeded >5% but less than 10%, corrected mortalities were calculated for all treated groups using Abbott's formula [19].

Significance in the spatial variations in corrected percentage mortality of *Aedes aegypti* and *Aedes albopictus* larvae and adults was evaluated using the General Linear Model (GLM) followed by Tukey's test for mean separation. Probit analysis (combined with log transformation) was used to determine the susceptibility status of *Aedes aegypti* and *Aedes albopictus* larvae to temephos in three studied areas. SPSS (version 23) was used for data analysis.

## 3. Results

### 3.1. Insecticide Susceptibility of *Aedes* Larvae

The highest percentage mortalities for *Ae. aegypti* larvae were observed at the highest temephos concentration (0.10 ppm) in Attanagalla (99.0 + 2.7%) and Negombo (95.0 + 2.7%), MOH areas. The lowest mortalities were recorded at 0.125 ppm concentration of temephos in the same localities (Table 1). According to the GLM, the percentage mortality rates of *Ae. aegypti* larvae denoted a significantly increasing trend along with the temephos concentration in both Attanagalla (*F*_1,4_ = 195.05, *P* < 0.001) and Negombo (*F*_1,4_ = 155.22, *P* < 0.001) MOH areas at 95% level of confidence. Further, *Ae. aegypti* larvae from the Attanagalla MOH area were significantly more susceptible to temephos than *Ae. aegypti* larvae of Negombo at all the concentrations (*F*_1,8_ = 9.532, *P* < 0.0001).

In *Ae. albopictus*, a gradual increase in percentage mortality was observed along with temephos concentration (Table 2). This trend was also found to be significant in all study areas (*F*_1,4_ > 44.91, *P* < 0.001). Further, statistics of GLM evidenced that the percentage mortality rates of *Ae. albopictus* larvae exposed to temephos varied significantly, among the three MOH areas (*F*_2,12_ = 15.73, *P* < 0.001). The highest susceptibility of *Ae. albopictus* larvae was observed in Attanagalla MOH area, which reported the 99.0 + 1.0% (98.0–100.0%) mortality at 0.375 ppm concentration of temephos. In both Dompe and Negombo, the highest percentage mortality rates were observed as 99.0 + 1.0% at 0.05 ppm (Table 2).

### 3.2. Determination of 24-Hour LC_50_ and LC_99_ for Exposed *Aedes* Larvae

The estimated LC_50_ and LC_99_ values of *Ae. aegypti* and *Ae. albopictu*s larvae (24-hour exposure period) against temephos from the three MOH areas, along with 95% Confidence Intervals (CI) retrieved from the Probit analysis, are indicated in Table 3. The lowest LC_50_ (0.020 ppm [0.018-0.023]) and LC_99_ (0.171 ppm [0.134-0.232]) values for *Ae. aegypti* larvae against temephos were observed from Attanagalla MOH area. In both Attanagalla and Negombo MOH areas, the LC_99_ values of *Ae. aegypti* for 24-hour exposure of temephos were 13.7 and 17.7 times higher than the WHO recommended concentration (0.0125 ppm) for 99% eradication of *Ae. aegypti* larvae, respectively.

In *Ae. albopictu*s larvae, the Dompe MOH area reported the lowest LC_50_ (0.008 ppm [0.006-0.010]) and LC_99_ (0.095 ppm [0.065-0.1710]) values. Similar to *Ae. aegypti* larvae, the LC_99_ values for 24-hour exposure of temephos were >4.6 times higher than the WHO recommended concentration (0.0125 ppm) for 99% eradication of *Ae. albopictu*s larvae (Table 3).

### 3.3. Insecticide Susceptibility of Adult *Ae. aegypti* and *Ae. albopictu*s Mosquitoes

**Figure 2:**
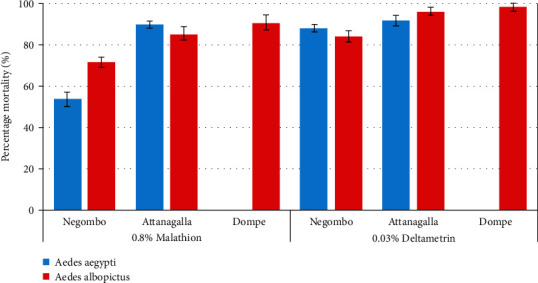
Percentage mortality rates of *Ae. aegypti* and *Ae. albopictus* females exposed to deltamethrin (0.03%) and malathion (0.8%) in the studied MOH areas.

The percentage mortality rates of *Ae. aegypti* and *Ae. albopictus* adult mosquitoes exposed to deltamethrin and malathion are depicted in Figure 2. The highest mortality rates of *Ae. aegypti* exposed to deltamethrin and malathion were observed in Attanagalla as 91.4 + 6.2% and 81.2 + 3.1%, respectively. Interestingly, only 53.1 + 8.2% of *Ae. aegypti* adults from Negombo were eradicated by malathion, denoting a relatively higher resistance (Figure 2). According to GLM, only percentage mortality rates of *Ae. aegypti* mosquitoes exposed to malathion denoted significant spatial variations (*F*_1,8_ = 77.78, *P* < 0.001).

The percentage mortality of *Ae. albopictus* mosquitoes exposed to deltamethrin for 24 hours varied significantly (*F*_2,12_ = 11.76, *P* = 0.01) at 95% level of confidence. *Ae. albopictus* mosquitoes from the Dompe MOH area denoted the highest percentage mortality against deltamethrin (0.03%) as 97.8 + 3.0%, while the lowest was observed from Negombo as 83.8 + 6.6% (Figure 2). A similar spatial variation was witnessed among *Ae. albopictus* mosquitoes exposed to malathion (0.8%), which also remained to be significant (*F*_2,12_ = 27.40, *P* < 0.0001). In this case, the highest mortality was observed from Dompe as 98.9 + 2.4%, while Negombo reported the lowest as 70.8 + 6.2%.

## 4. Discussion

Dengue vector control strategies largely depend upon the use of larvicides in the breeding sites to target the vectors at the immature stages in their life cycle and space sprays aiming at the adult infective stages of the potential dengue vectors [20]. The development of insecticide resistance is a major threat to public health vector control measures all around the globe [21], including Sri Lanka [10, 14, 22]. However, there are limited published data on insecticide susceptibility. Spatial heterogeneity of insecticide resistance could have important implications for vector control efficacy, particularly when vector control strategies are designed to be applied across a large geographical area. The development of scientifically sound vector control and insecticide resistance management strategies for dengue vectors depends on the patterns and drivers of spatial heterogeneity in insecticide resistance [23]. The present study revealed the development of resistance to commonly used larvicide and adulticides among both the *Ae. aegypti* and *Ae. albopictus* population in Gampaha District, Sri Lanka.

According to the recommendation of the WHO, temephos 1% sand granules and temephos 50% EC are the most suitable and approved larvicides for container breeders worldwide [24, 25]. In Sri Lanka, temephos 1% sand granules are mainly recommended for *Aedes* control in domestic water storage containers that cannot be destroyed and covered with a lid and temephos 50% EC promotes for large scale breeding grounds such as abandoned boats, concrete slabs, construction sites, and yards with machinery parts especially during dengue epidemics [13]. The efficacy of the larviciding effect of temephos is mainly dependent on the frequency of application, contact period, application dosage, and frequency of usage. However, it is noteworthy that the overuse of chemicals at higher doses facilitates the mosquito-resistant onset [26].

The present study indicated a higher prevalence of temephos resistance among both *Ae. aegypti* and *Ae. albopictus* in line with previous studies done in Sri Lanka and other countries. [24, 26–29]. The larval mortality rates for temephos were ranged from 14 to 36% for *Ae. aegypti* and 50-78% for *Ae*. *albopictus* for the WHO discriminating dosage during the study. Based on the larval bioassay results, the highest temephos resistance for *Ae. aegypti* was observed from urban areas in Negombo which contributed to the highest number of dengue cases reported in the Gampaha District during the last five years [6]. This result was not that surprising due to the widespread and frequent application of insecticides for larval control activities in the area. Therefore, it could be stated that frequent applications of insecticide in urbanized areas have induced the resistance in *Aedes* population [24]. However, the continuous application of temephos in these areas may create untoward effects in the current vector control programmes. Hence, the application of the temephos chemical for vector control should be rotated with another alternative chemical group to delay the resistance development by the mosquitoes against the temephos.

A low level of resistance was observed in *Ae. albopictus* compare to *Ae. aegypti* especially in rural and suburban areas in Gampaha District in line with the previous studies [24, 30]. It can be stated that past efforts to control malaria epidemics may have contributed to the development of resistance among *Aedes* populations in rural settings. Chemical larviciding with temephos was first introduced in Sri Lanka as a supplementary malaria control measure in 1997, and still, it is continued to be used from time to time for malaria outbreak control activities within the country simultaneous with dengue [31]. In addition to the usage of chemicals in health programmes, the agriculture sector also utilizes a vast array of chemicals in which the usage in the health sector could be negligible. The LC_99_ for *Ae. albopictus* was highest in the Dompe MOH area which represents a rural setup.

In Sri Lanka, the Gampaha district has an ideal climatic and ecological environment for the cultivation of pineapple. Therefore, pineapple plantation is being continued commercially targeting both the local and foreign markets [32]. Therefore, agricultural use of organophosphate insecticides in pineapple plantations could be a reason for this higher level of resistance for temephos in this region. Hence, the present study warrants the need for a common regulating body for insecticide management integrating both public health and agricultural pest control activities and also to improve the knowledge of public health staff on integrated vector management including source reduction and biological vector control strategies.

In Sri Lanka, mostly, thermal fogging is conducted with pyrethroid insecticides, especially pesguard FG161 and occasionally with technical malathion on a rotational basis for the control of adult vector densities [13]. Recent studies have demonstrated either development of resistance or a decrease in the susceptibility to synthetic insecticides in *Aedes* mosquitoes in many counties in the world [28, 33, 34]. The present study also revealed the presence of resistance among *Ae. aegypti* populations for both currently used organophosphate and pyrethroid insecticides in the Gampaha District, Sri Lanka.

Malathion resistance has been widely reported in both *Ae. aegypti* and *Ae. albopictus* mosquitoes in Sri Lanka [10, 14, 24]. The results of the current study also suggest that technical malathion has low efficacy in controlling both *Ae. aegypti* and *Ae. albopictus* mosquitoes especially in urban and suburban areas, where it has demonstrated a high level of resistance to the insecticide. The resistance to malathion might have been developed over time due to prolonged and frequent use in public health programmes during disease outbreaks situations [35]. The present study also indicates an emerging resistance to malathion in the Dompe area as the mortality obtained was below 97% for *Ae. albopictus* mosquitoes. Similarly, adult bioassay results of deltamethrin showed that tested mosquito species have also developed resistance to pyrethroid insecticides in the Gampaha District which is also associated with the extensive and routine application of pyrethroid in dengue control activities and increasing household use of pyrethroids.

This study found that in all three localities, *Ae. aegypti* were more resistant than *Ae. albopictus* for both pyrethroid and organophosphate insecticides. The high prevalence of resistance in *Aedes aegypti* could be due to high selective pressure with the frequent application and higher exposure to insecticide either during fogging by the health and local authorities. Since *Ae. aegypti* is an endophilic mosquito that prefers to rest and breed indoors [36], it is more likely to be exposed to household insecticides than *Ae. albopictus.* The emergence of a high level of resistance to both the currently used insecticide categories may create difficulty in determining suitable insecticides for dengue control and challenges in future vector control approaches. Most importantly, the development of resistance among *Ae. albopictus* population against pyrethroids insecticides is a major threat within the region since it is the predominant dengue vector in most of the suburban and rural areas of the country and pyrethroids are the most popular insecticide class all around the globe including Sri Lanka [14, 37].

The results of this study warrant the vector management authorities on the proper application of insecticides and rational use in vector control. Therefore, the susceptibility status of vector mosquitoes should be continuously monitored especially in dengue-endemic areas parallel to the routine surveillance programme. Therefore, the application of chemicals in public health vector control programmes should be carefully evaluated, and the insecticide resistance management system should be implemented with the collaboration of the entire vertical vector campaigns.

The study also indicates the importance of a molecular approach to determine the emergence and occurrence of Knockdown Resistance (kdr) mutations among *Aedes* populations due to the widespread use of pyrethroid chemicals to implement a successful insecticide resistance management programme in the country.

## 5. Conclusion

Variation in the resistance levels to the insecticides by the dengue vectors in different areas would be a challenging task for routine insecticide-based vector control approaches. Therefore, it should be allied with the susceptibility levels to insecticides by mosquito vectors. Further, the rational use of insecticides for vector control is a high priority. The present study warrants a common regulating body for insecticide management in conjunction with public health and agricultural pest control activities.

## Figures and Tables

**Table 1:** Percentage mortality rates of *Ae. aegypti* larvae exposed to different concentrations of temephos.

Concentration/ppm	Percentage mortality (%)	
	Attanagalla	Negombo
0.125	36.0 + 1.9 (34.1–37.9)	14.0 + 1.8 (12.2–15.8)
0.250	51.0 + 4.5 (46.5–55.5)	21.0 + 2.4 (18.6–23.4)
0.375	64.0 + 1.9 (62.1–65.9)	36.0 + 1.9 (34.1–37.9)
0.050	89.0 + 1.9 (87.1–90.9)	70.0 + 4.2 (65.8–74.2)
0.100	99.0 + 1.7 (97.3–100.0)	95.0 + 2.7 (92.3–97.7)

**Table 2:** Percentage mortality rates of *Ae. albopictus* larvae exposed to different concentrations of temephos.

Concentration (ppm)	Percentage mortality (%)		
	Attanagalla	Dompe	Negombo
0.0625	45.0 + 2.7 (42.3-47.7)	49.0 + 3.6 (45.4-52.6)	10.0 + 4.2 (5.8–14.2)
0.0125	78.0 + 2.5 (75.5–79.5)	58.0 + 3.0 (55.0-61.0)	50.0 + 2.2 (47.8–52.2)
0.250	85.0 + 2.7 (82.3–87.7)	68.0 + 2.5 (65.5–70.5)	64.0 + 1.9 (62.1–65.9)
0.375	99.0 + 1.0 (98.0–100.0)	80.0 + 3.5 (76.5–83.5)	85.0 + 1.6 (83.4–86.6)
0.05	100.0 + 0.0	99.0 + 1.0 (98.0-100.0)	99.0 + 1.0 (98.0–100.0)

**Table 3:** LC_50_ and LC_99_ values of *Ae. aegypti* and *Ae. albopictus* larvae exposed to 24 hours for temephos.

Species	Study area	LC_50_ (ppm)	LC_99_ (ppm)	Resistance factor
*Aedes aegypti*	Negombo	0.0381 (0.036–0.042)	0.221 (0.181–0.280)	17.7
	Attanagalla	0.020 (0.018–0.023)	0.171 (0.134–0.232)	13.7
*Aedes albopictus*	Negombo	0.015 (0.014–0.017)	0.088 (0.073–0.110)	7.0
	Attanagalla	0.007 (0.006–0.008)	0.058 (0.046–0.076)	4.6
	Dompe	0.008 (0.006–0.010)	0.095 (0.065–0.1710	7.6

## Data Availability

All data are available from the authors and will be provided upon reasonable request.

## Abbreviations

EC: Emulsifiable concentrate
GLM: General Linear Model
Kdr: Knockdown Resistance
LC: Lethal concentration
MOH: Medical Officer of Health
WHO: World Health Organization.
